# Humans in the upstream can exacerbate climate change impacts on water birds’ habitat in the downstream

**DOI:** 10.1038/s41598-021-99822-8

**Published:** 2021-10-12

**Authors:** Saeideh Maleki, Vahid Rahdari, Nicolas Baghdadi

**Affiliations:** 1 Department of Environment, Faculty of Natural Resources, University of Zabol, Zabol, Iran; 2 INRAE, TETIS, University of Montpellier, Montpellier, France

**Keywords:** Ecology, Ecosystem ecology

## Abstract

The present paper aims to quantify how human-made changes in the upstream exacerbate climate change impacts on water birds’ habitat in the downstream. To reduce climate change effects and design adaptation policies, it is important to identify whether human activities understate or overstate the effects of climate change in a region on its inhabitants. This paper also shows how human activities may magnify climate change impacts both locally and regionally. Land-use/land-cover change as the important sign of human-made destruction in an ecosystem was detected in the upstream of the Helmand basin over 40 years. Owing to conflicts in Afghanistan, studies on this basin are rare. The water bird’s habitat suitability maps during the study period were created using the maximum entropy model and the multi-criteria evaluation method. The post-classification method was applied to show the land-use/land-cover change over 40 years. These results were compared to the area of suitable habitat for water birds. The findings of these analyses indicated that the irrigated farming was expanded in the upstream despite climate change and water limitation, while the water birds’ habitat in the downstream was declined. These results revealed that the unsustainable pattern of farming and blocking water behind dams in the upstream exacerbated the negative effects of climate change on water birds’ habitat in the downstream. The significance of this study is to demonstrate the role of human in exacerbating climate change impacts both locally and regionally.

## Introduction

Nowadays, the global community has sufficient evidence about the causes of climate change and its negative effects on all inhabitants. Different studies have described the role of human in occurring climate change^1–3^. Undoubtedly, reduction of the factors causing climate change is an important solution to protect our earth for our descendants^4,5^, but protection of the ecosystems currently dealing with climate change is important as well. Although in some parts of the world, governments and decision-makers design conservational plans to minimize the destructive effects of climate change on ecosystems^6,7^, sufficient attention is not paid to this issue in otherregions^8–10^.Unsustainable land-use policies increasing the water consumption in arid regions^11,12^, and deforestation with extreme floods can be among these destructive activities^13,14^. Furthermore, policies considering the combined effects of human activity and climate change can considerably contribute to develop climate change adaptation policies^15–18^. However, to protect the ecosystems encountering climate change, it is crucial to highlight the role of the human in exacerbating the negative effects of climate change. Fu et al.^19^ maintained that land-use/land-cover and climate change affected the ecosystem functions by degradation patterns and processes. Gilliani et al.^20^ investigated the irrigated agriculture affected by climate change and the behaviors of groups of farmers to mitigate the drought effects. This study emphasized the role of irrigating policies in reducing the negative effects of climate change on food production. Willson et al.^21^ argued that to develop effective strategies in order to minimize human-wildlife conflict, we must understand the relative influences that climate change and humans have on wildlife habitats. According to Mukul et al.^22^, new policies are required to conserve the Bengal tiger against the climate change effect. Reduction of large-bodied frugivorous due to habitat disturbance^23^ and hunting^24^ and loss of functionally specialized species^25^ were reported as the results of land-use change. Bender et al.^26^ stated that climate change could have similar effects on functionally specialized species. In addition, it is worth to investigate whether human effects locally interact with climate change consequences or they affect the far region. Munia et al.^27^ stated that uncontrolled land and water development in upstream regions could escalate the risk of water variability in downstream regions^14,28,29^. Concerns about water variability are one of the most important issues for conflicts overshared water basins^30^.

This paper aims to investigate if human activities in the upstream of a basin can exacerbate the effects of climate change on habitats in the downstream and to highlight the importance of the human in aggregating the consequences of climate change on water birds’ habitat. This study was conducted in the Helmand basin, where the previous research confirmed the occurrence of climate change^31^, leading to a significant reduction in precipitation and an increase in mean annual temperature^31,32^. Since, in the downstream of this basin, there are wetlands providing the most important habitat for migrant, resident, and wintering water birds in a vast desert, it is important to identify the effects of humans on the habitat of water birds. To reach this goal, land-use/land-cover maps of the upstream of the Helmand basin were created using remote sensing techniques. The water birds’ habitat suitability in the downstream was mapped, and its changes were detected within 40 years. The habitat suitability changes were investigated in terms of land-use and land-cover changes in the upstream of the watershed. The assumption in this study is that if land-use/land-cover change in accordance with the water limitation and habitat suitability alteration, humans do not exacerbate the climate change effects. However, if land-use classes with high water demand are developed after occurring climate change and water limitation and the alteration of land-use development is in contrast to water birds’ habitat suitability, humans aggregate the climate change effects. The significance of this study is to demonstrate the role of human in exacerbating climate change impacts both locally and regionally. The evidence is crucial for global community to be informed about humans’ aggregating effects. In addition, the study area is a share international water basin on which studies are rare due to conflicts in Afghanistan.

## Results

**Figure 1:**
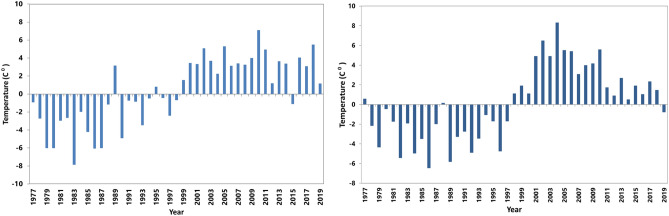
Precipitation anomalies for (**a**) Kandahar, (**b**) Zabol.

**Figure 2:**
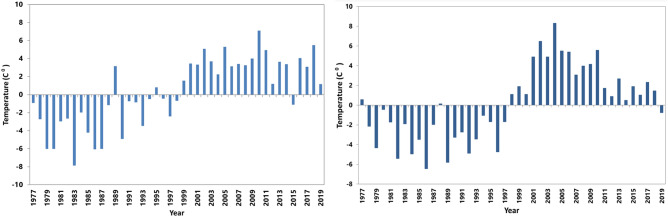
Mean annual temperature anomalies for (**a**) Kandahar, (**b**) Zabol.

The results of mean annual precipitation and temperature anomalies are presented in Figs. 1 and 2, respectively. Figure 1 shows normal precipitation before 1980 and an increase in precipitation by the end of the '80 s. It shows a reduction in precipitation by the end of the '90 s. Figure 2 shows an increase in mean annual temperature. Therefore, the climate of the study area is getting warmer. These results showed the climate is getting warmer and drier.

**Figure 3:**
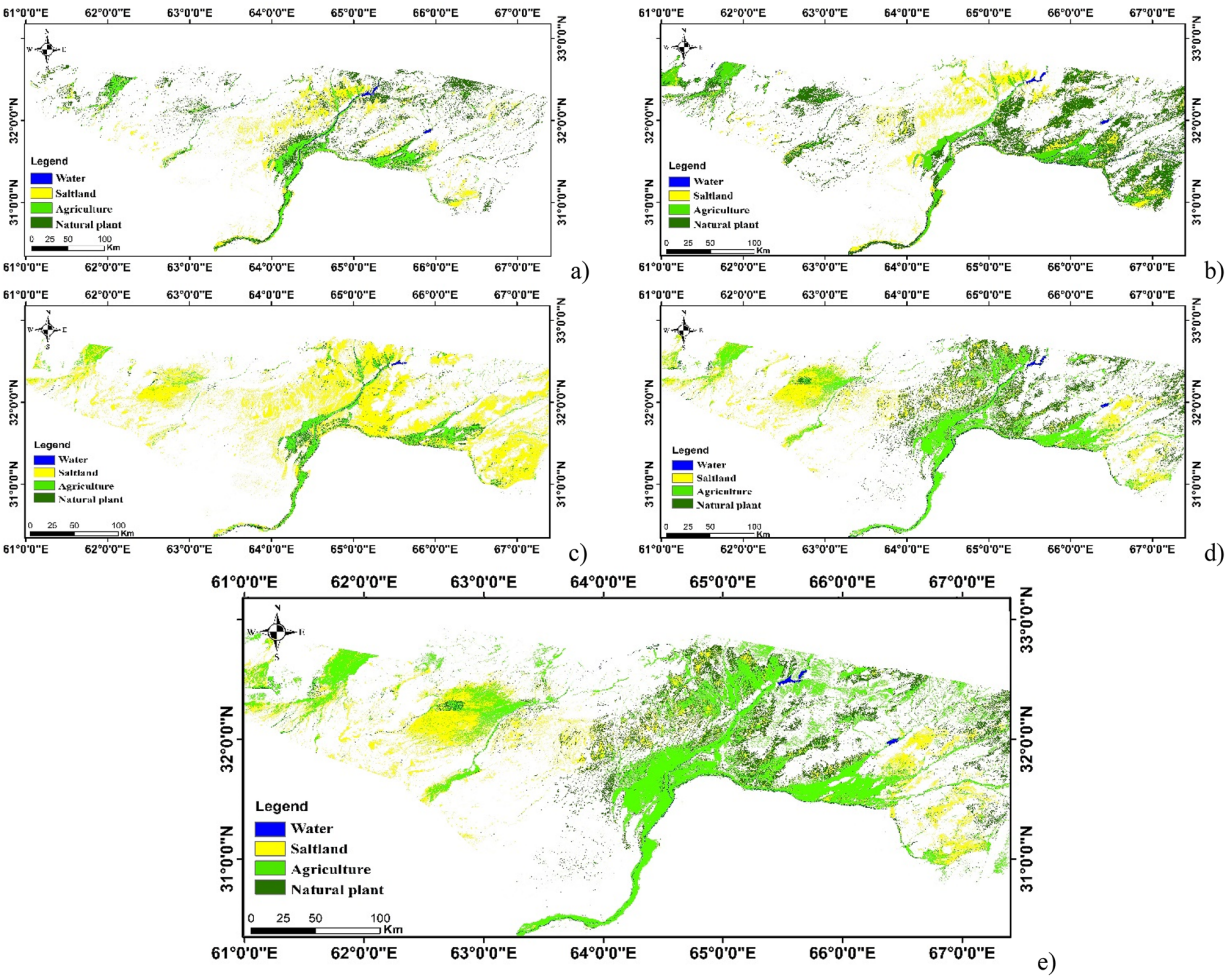
Land-use/land-cover of upstream (**a**) 1977, (**b**) 1991, (**c**) 2002, (**d**) 2014, (**e**) 2018. ( *Source*: The maps were created by QGIS 3.16: https://qgis.org/en/site/forusers/download.html and ArcGIS 10.5.1: https://www.esri.com/en-us/home).

The land-use/land-cover maps of the upstream of the Helmand river basin were created for 1977, 1991, 2002, 2014 and 2018 (Fig. 3a–e). Table 1 shows the results of the accuracy assessment of the maps. Table 2 illustrates the confusion matrix of land-use/land-cover map annually. As seen, the produced maps have acceptable accuracy, and they are accurate enough to be used^33,34^ (82%, 85%, 86%, 90% and 89% for 1977, 1991, 2002, 2014 and 2018, respectively). The area of main land-cover classes, including agriculture, water body and natural plants were calculated using the produced maps.

**Table 1:** Overall classification accuracy and kappa statistics.

	1977	1991	2002	2014	2018
Kappa	0.80	0.82	0.84	0.88	0.88
Overall accuracy	82%	85%	86%	90%	89%

**Table 2:** The confusion matrix of Land-use/ land-cover of upstream (a) 1977, (b) 1991, (c) 2002, (d) 2014, (e) 2018.

Land cover class	Reference data			
	Water	Saltland	Agriculture	Natural plants
**1977 map data**				
Water	82.8	1.8	6.8	8.6
Saltland	3.4	84.2	5.5	6.9
Agriculture	2.6	3.3	80.3	13.8
Natural plants	1.7	4.8	12.7	80.8
**1991 map data**				
Water	87.5	2.1	3.8	6.6
Saltland	1.3	86.2	5.8	6.7
Agriculture	2.4	2.5	83.3	11.8
Natural plants	1.5	2.9	10.8	84.8
**2002 map data**				
Water	89.8	2.1	3.5	4.6
Saltland	1.5	87.8	5.3	5.4
Agriculture	1.1	7	82.8	9.1
Natural plants	0	3	13.3	83.7
**2014 map data**				
Water	90.8	1.4	3.2	4.6
Saltland	1.9	91.1	4.6	2.4
Agriculture	1.4	4.2	89.1	5.3
Natural plants	2	1.4	7.3	89.3
**2018 map data**				
Water	88.4	2.4	3.8	5.4
Saltland	1.8	90.8	4.7	2.7
Agriculture	1.2	3.1	89.9	5.8
Natural plants	2.4	1.1	8.3	88.2

**Figure 4:**
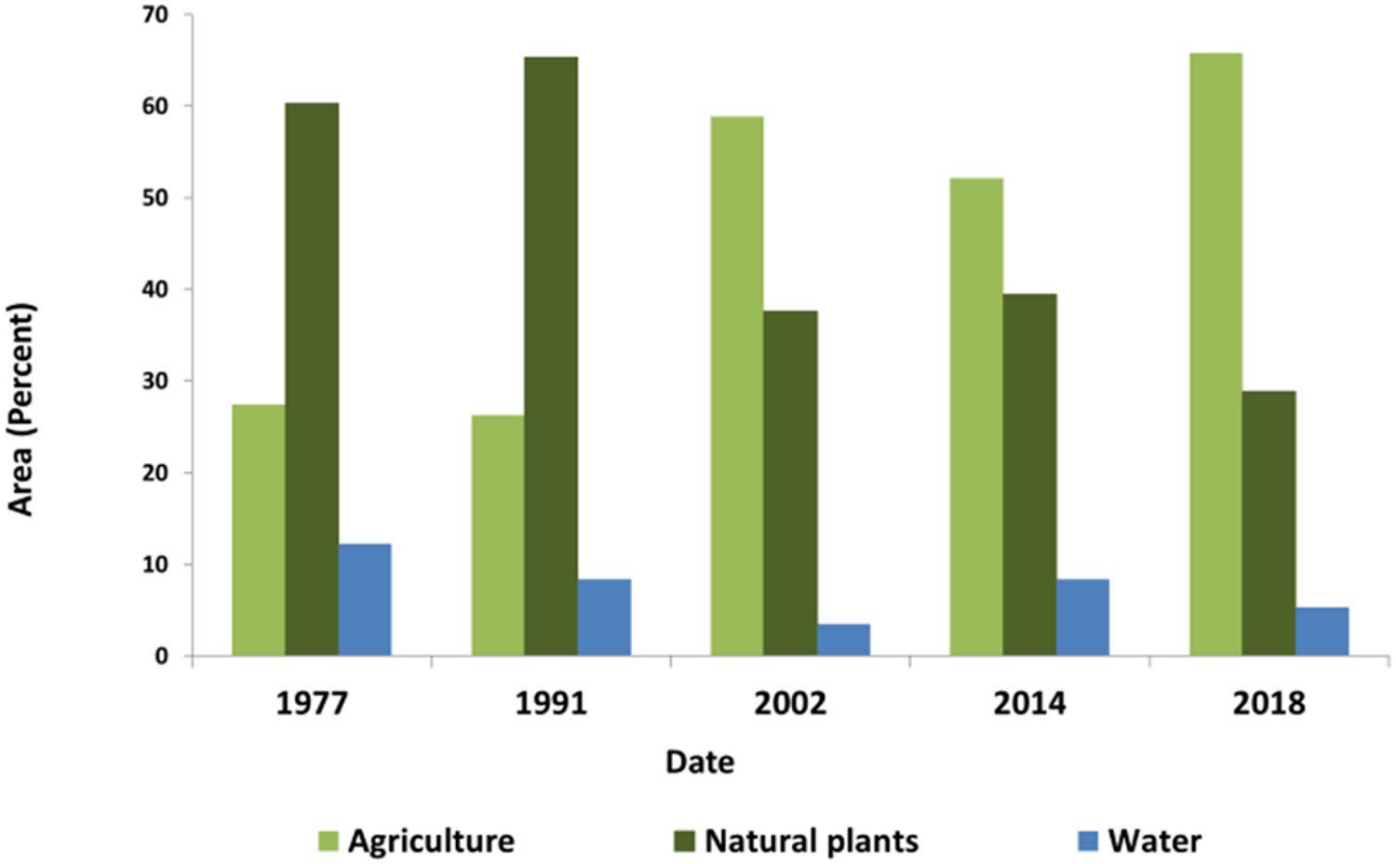
The percentage of land-use/land-cover area in the upstream of the Helmand river basin.

For a better interpretation, the area of land-use/land-cover classes was presented as percentage in Fig. 4. This figure shows that under normal conditions (1977), natural plants are the dominant class covering approximately 70% of the Helmand river basin. In 2002, 2004 and 2008, the area of natural plants fell to 38%, 40% and 30%, respectively, being normal after a significant decline in precipitation and an increase in temperature. But, as seen the area of agriculture developed under drought conditions in 2018 and it changed to the nearly 70% in this year that is the dominant land-cover, while the area of agriculture class in normal and flood condition (1977 and 1991 respectively) was below 30%. These results show considerable development in the agricultural area in the upstream of this basin.

**Figure 5:**
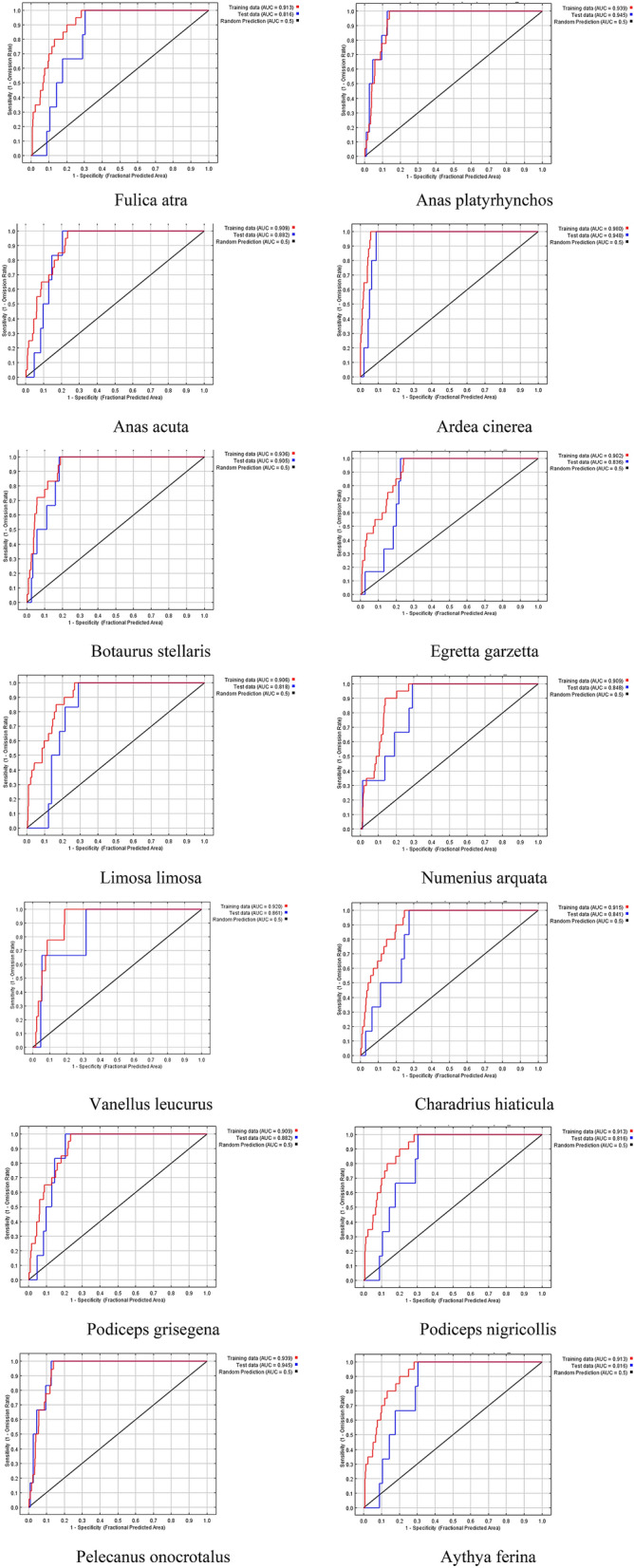
The Receiver Operating Characteristic (ROC) curve of the habitat suitability map for each species in Table 4. The AUC value 0.5 indicates that the model performance is not better than that of random, while values closer to 1.0 indicate better model performance.

In this paper, the habitat suitability map of 2014 was created by Maxent. The graph of the Area Under the Receiver Operating Characteristic (ROC) Curve or AUC was used to evaluate the model. The AUC value of 0.5 indicates that the performance of the model is not better than that of random, while values closer to 1.0 indicate better model performance. Figure 5 presents the ROC curve of the habitat suitability map for each species in Table 4. The AUC values are higher than 0.9. Therefore, the model performance for all species is acceptable.

**Figure 6:**
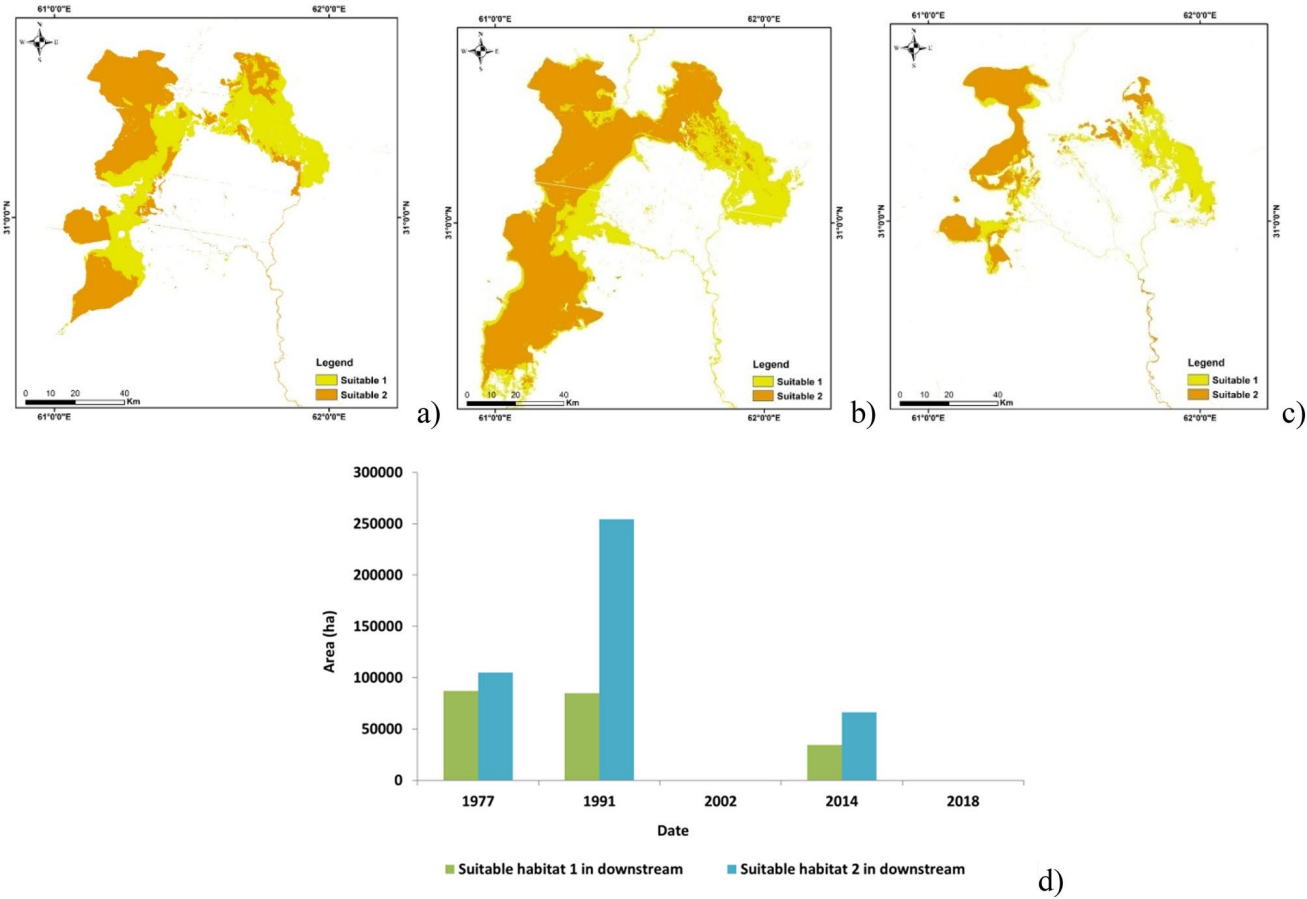
Water birds’ habitat suitability map in the downstream: (**a**) 1977, (**b**) 1991, (**c**) 2014, (**d**) the area of a suitable habitat (in 2002 and 2018, the wetlands were dry, and the area of water birds’ habitat was zero). ( *Source*: The maps were created by QGIS 3.16 https://qgis.org/en/site/forusers/download.html and ArcGIS 10.5.1 ).

Water bird’s habitat suitability map in the Helmand river basin downstream was generated for 1977, 1991, 2014 (Fig. 6). In 2002 and 2018, the wetlands were dry and no suitable habitats existed for water birds in the Hamoun wetlands. Figure 6d presents the calculation of the area of both suitable 1 and 2 classes. As seen, the largest suitable habitat was provided in 1991. Regarding this fact that this habitat is located within a desert in an arid area, a habitat with approximately 350,000 ha is valuable for water birds. It is the importance of these wetlands to select as the wetlands with international importance^35^. The area of the suitable habitat fell dramatically after 1991 so that in 2002 and 2018 no suitable habitat existed for water birds in the downstream. Considering this fact that the Ramsar convention selected a large part of the Hamoun wetlands in the downstream as a protected area owing to its importance for water birds^35^, these results show the intensive negative pressure on these birds due to considerable habitat degradation.

## Discussion

**Figure 7:**
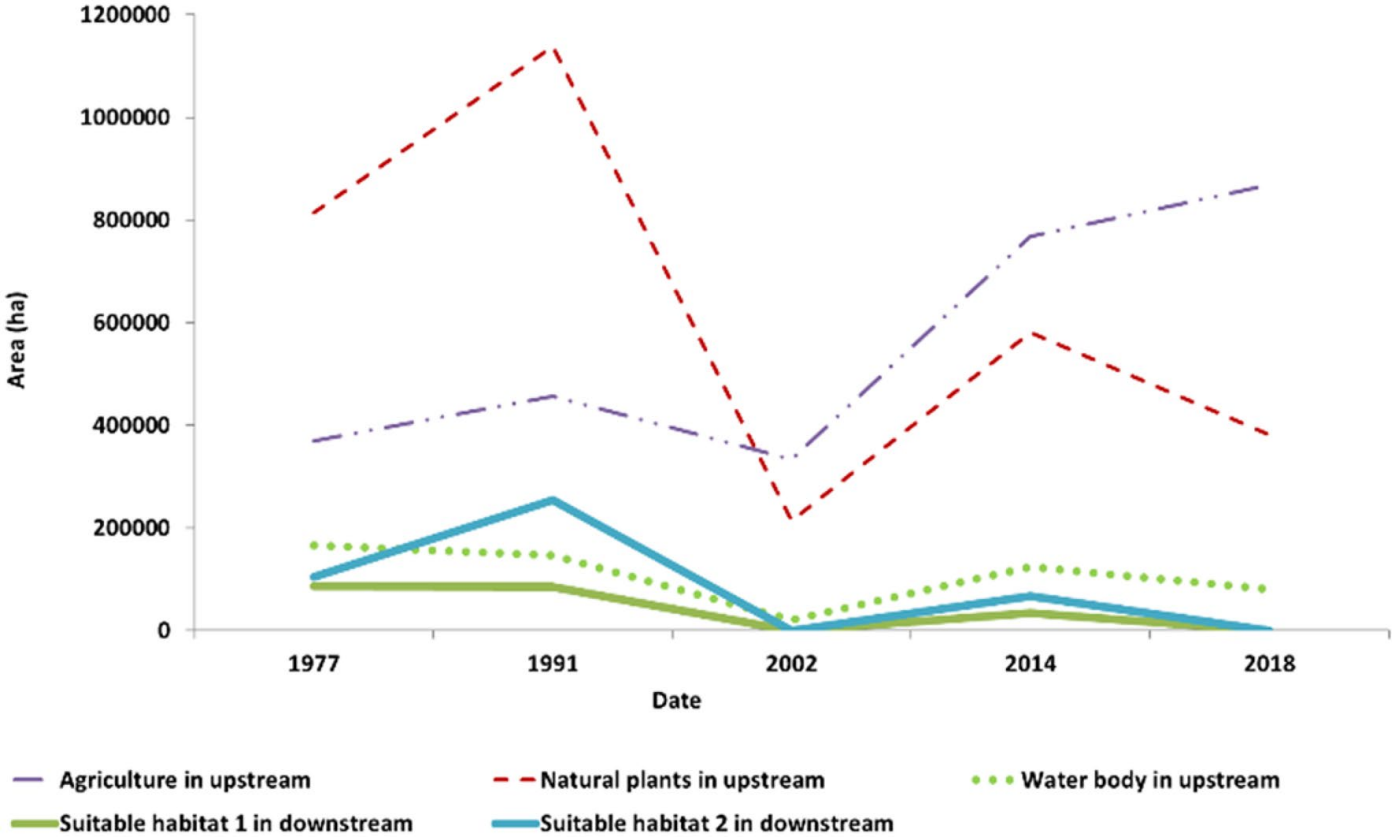
Comparison between the area of main land-cover classes in the upstream and suitable habitat in the downstream.

Figure 7 presents the comparison between the area of the main land-cover classes in the upstream and the suitable habitat in the downstream. The figure reveals that in 1977 and 1991 natural plants were dominant cover in the upstream of the basin. The results of climate variability analysis (Figs. 1, 2) mentioned these years with normal and high precipitation, respectively. Lumbroso^36^ reported large floods in 1991 in the downstream. In all years after 1991, natural plants were decreased. Figures 1 and 2 showed the climate is getting warmer and drier. Maleki et al.^31^ confirmed that the climate change occurred in the last years of the 90th, leading to precipitation decline and temperature increase; therefore, the decline of natural plants is due to precipitation decrease. In 2002, agriculture was dominant, while natural plants were decreased dramatically. Agriculture did not decrease in the upstream in 2002, while wetlands and water bird’s habitat nearly vanished. In 2014, all land-covers developed; however, the increasing rate of agriculture was higher than that of other land-covers, so that the area of agriculture in the upstream was higher than those of 1977 and 1991 when no precipitation decline existed. As seen, in 2014, the area of water birds’ habitat and water body were smaller than those of normal years. In 2018, the water bird’s habitat vanished completely due to drying-up of the wetlands in the downstream, while the agriculture area was higher than that in all years mentioned in the study period. FAO Technical Cooperation Programme^37^ and the study of Goes et al.^32^ mentioned farming as high water demand land-use in this region. Furthermore, since there is no natural lake in the upstream, and there are two dams in this region, the area of water body in the upstream in Fig. 7 reflects the water behind the dams. As seen, the area of lakes behind the dams in 2018, when the wetlands were dry, was near to those of 1991 and 2014, when wetlands were not dry (The area of water body was 145,809 ha in 1991, 123,545 ha in 2014, and 11,576 ha in 2018). Blocking this volume of water behind the dam in the upstream when the wetlands are dry and the dramatic increase in irrigated farming are the negative effects of humans aggregating the water limitation after the decline of precipitation in the last years of the 90th. The current policy in the upstream is concentrated on the irrigated farming consuming a huge amount of water^32,37^. This policy not only threats the water bird’s habitat downstream, but also is not sustainable in the condition of prolong drought in this basin. Therefore, farmers in the upstream must replace their cultivating strategies with methods minimizing the water demand in order to avoid crop failure and increase the food production. This change in land-use policies were applied in other regions with the same condition, providing a better life for human and wildlife (e.g., El-Khoury et al.^9^; Van der Pol et al.^10^; Scheraga et al.^38^; Langerwisch et al.^39^; Scullion et al.^40^; National Research Council^41^).

In conclusion, the previous research in the Helmand basin confirmed that the occurrence of climate change in this basin led to a significant reduction in precipitation and an increase in mean annual temperature. The effect of human on water bird’s habitat affected by climate change was investigated using land-use/land-cover change detection of the Halmand basin over 40 years. This study shows that in an arid area where climate change causes water limitation, humans can intensify the effect of climate change on water birds. Development of agriculture area with high water demand and application of unsustainable irrigating methods and blocking of the water behind the dam in the upstream without considering the downstream are the human activities leading to drying out the wetlands in the downstream and water birds’ habitat degradation, thereby exacerbating the negative effects of water limitation on water birds.

By considering this fact, local people are dependent on the farming to promote sustainable development for this region, by supporting the life of human communities in the longer time and protecting the natural ecosystem. Thus, it is necessary to reconsider the current farming pattern. In this basin, where the precipitation was decreased and the temperature was increased, irrigation methods with lower water demand are preferred.

This study revealed that human effects in the upstream can aggregate the effect of climate change. Land-use policies and hydrological management in the upstream increased the water limitation in the downstream, leading to complete degradation of water bird’s habitat in some years and loss of suitable habitat in other years.

## Material and methods

### Study area

**Figure 8:**
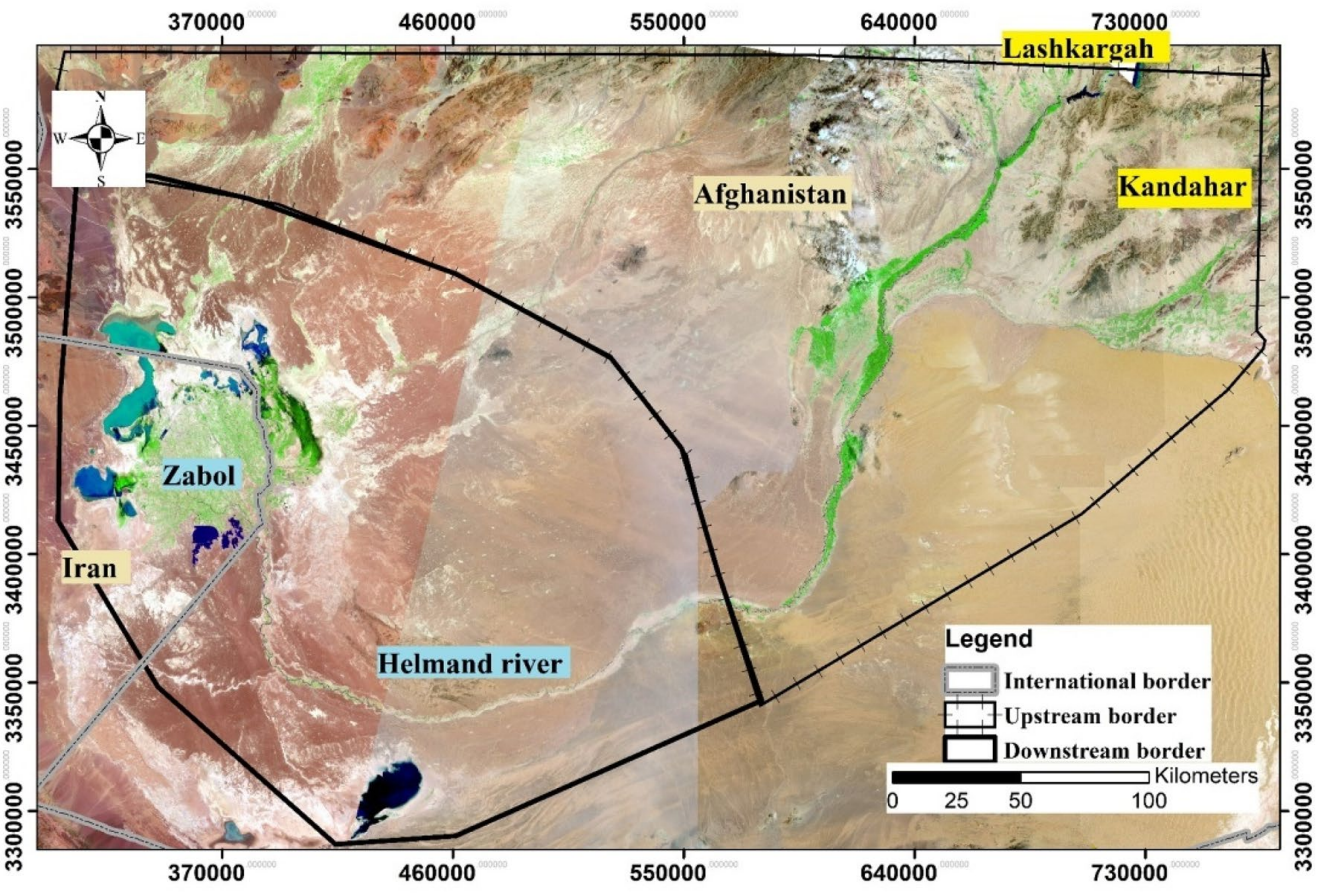
Helmand river basin. (*Source*: the satellite image was downloaded freely from www. https://earthexplorer.usgs.gov and processed by QGIS 3.16 https://qgis.org/en/site/forusers/download.html and ArcGIS 10.5.1 https://www.esri.com/en-us/home).

The study area is the Helmand river basin. The total area of this basin is approximately 400,000 km^2^. It is located in southern Afghanistan (81.4% of the basin), eastern Iran (15%) and northern Pakistan (3.6%) (Fig. 8). The highest elevation in the basin is over 4400 m above sea level (masl) in the Hindu Kush Mountains to 490 masl in the Sistan plain^32^. The Helmand River feeds the Hamoun wetlands at the end tail of this basin. These wetlands are the most important habitat for migrant, resident and wintering water birds in the midst of a vast desert. Accordingly, a large part of these wetlands are listed as a biosphere reserve^35^. The previous studies in the Helmand River basin confirmed the occurrence of climate change^31^, leading to a significant reduction in precipitation and an increase in the mean annual temperature^31,32^. LashkarGah and Kandahar in Afghanistan are the main cities in the upstream of the Helmand River basin. Ghazni, Musa Qala and Arghandab rivers join the Helmand River upstream in LashkarGah. The Helmand River then follows over 400 km through the desert in Nimrooz Province and up to the Iranian border in the downstream. This river feeds the Hamoun wetlands in the downstream of this basin^42^.

### Climate variability analysis

The mean annual precipitation and temperature anomalies were calculated using Eq. (1).

1 $$P_{yanomaly} \left( {i,j} \right) = \frac{{Py{-}\left[ { \, Mean\;P\left( {i,j} \right)} \right]}}{{STD\left( {P\left( {i,j} \right)} \right)}}$$

*MeanP(i,j)* = Mean of study period, Py = Measurement from year (y), STD = Standard deviation of study period.

### Land-use/cover mapping

To create land-use/land-cover maps, Landsat Multi Spectral Scanner (MSS) images of 18 June1977, Landsat Thematic Mapper images (TM) of 15 June 1991 and 13 June 2002, the Operational Land Imager (OLI) of 13 May 2014 and 25 June 2018 were applied (Table 3). These images were freely downloaded from the U.S. Geological Survey (USGS) website (www.https://earthexplorer.usgs.gov). In addition, the Google Earth™ (http://earth.google.com), the FAO project’s land-use map of Afghanistan^37^, the results of Maleki et al.^43^ and field measurements within the Iranian borders were used.

**Table 3:** Data used in this study.

Data	Scene	Spatial resolution	Dates of images/data used (the first scene)
MSS	7 scenes	60 m	18/06/1977
Landsat 4–5	7 scenes	30 m	15/06/1991
Landsat 4–5	7 scenes	30 m	13/06/2002
Landsat8–OLI	7 scenes	30 m	13/05/2014
Landsat8–OLI	7 scenes	30 m	25/06/2018
Field measurements		–	May 2014 and June 2018
FAO project’s land-use map of Afghanistan			2015
Google Earth		Varying resolution	
Topographic map (Iran National Cartographic Center, 1949 and 2009)		1:50,000	1949 and 2009

For land-use/land-cover mapping, the field campaign was conducted based on the satellite overpass. A total of 185 samples within the land-cover classes were collected based on the stratified random sampling method. Stratified classes were determined based on land-cover classes in this region. All the points were randomly recorded in the homogeneous area to omit the effect of neighboring classes^44^. All the points were registered using the global positioning system (GPS). These samples were used as training samples in image classification and validation.

Changes in human activities in the upstream was detected using land-use/land-cover maps dating back to1977, 1991, 2002, 2014, and 2018. Water body, agriculture area, natural plants and salt land are the main land-cover classes in this region. The training polygon for each land-use/land-cover class was selected using field measurements in Iran and previous studies^31,37,43,45^. Each image was classified using the support vector machine (SVM) method. In this algorithm, training samples are applied to obtain a linear separating hyperplane in a multi-dimensional feature space. The SVM algorithm works based on minimizing the error risk and maximizing the margins between the separating hyperplane and the closest training samples^46^. Totally, 70% of the field samples were used in the training process to classify the satellite images. Radial basis function was selected as the Kernel function. In cases of non-linearly separable data, a kernel function was used for non-linear transformation of the input data^47^.

Accuracy assessment was conducted on each land-use/land-cover map using 30% of the sample as validation points not used in the training step (the remaining 54 points). The validation points for 2014 and 2018 were achieved using field measurements. For 2002, the validation points were extracted from the Google Earth and other reference materials. The 1977 and 1991 classified Landsat images were compared to the 1986 topographic maps. The overall accuracy and the kappa coefficient (k) were calculated for all maps.

### Species distribution modelling

In the Helmand basin, the Hamoun wetlands are the most important habitat for water birds^43^. Since the wetlands were dry in 2018, we used the data of a previous study^43^. To collect the data, the wetlands were monitored for the water birds’ nesting and resting habitats in May 2014. In this regard, the methods used by Gregory et al.^48^ were applied to record the data of water birds. Based on this method, water birds were monitored in point transects using telescopes (15 × 60) and binoculars (10 × 40) for 10 min at each point. Additionally, their nests were investigated through 100 m line transects (Gregory et al.^48^). Stratified random sampling method was used to determine the starting points. Direction of transects was set on the left of the starting point. The locations of selected nests were recorded using the Global Positioning System (GPS). Totally, 122 points of water bird’s occurrence were recorded. At each point, the number of water birds was recorded. Table 4 presents the total number and names of these water birds at all points. Furthermore, water depth, landscape type, vegetation height and vegetation cover percentage were recorded as habitat factors.

**Table 4:** The total number and names of water birds listed at 122 observation points and AUC of habitat model for each species.

Order	Family	Species	Total number of water birds		AUC
Anseriformes	Anatidae	*Aythya ferina*	150		0.91
		*Anas platyrhynchos*	352		0.93
		*Anas acuta*	30		0.90
Gruiformes	Rallidae	*Fulica atra*	420		0.91
Pelecaniformes	Ardeidae	*Ardea cinerea*	157	500	0.98
		*Egretta garzetta*	98		0.90
		*Botaurus stellaris*	245		0.93
	Pelecanidae	*Pelecanus onocrotalus*	100		0.93
Charadriiformes	‏Scolopacidae	*Limosa limosa*	270	600	0.90
		*Numenius arquata*	126		0.90
	Charadriidae	*Vanellus leucurus*	10		0.92
		*Charadrius hiaticula*	194		0.91
Podicipediformes	Podicipedidae	*Podiceps grisegena*	115	200	0.90
		*Podiceps nigricollis*	85		0.91

**Figure 9:**
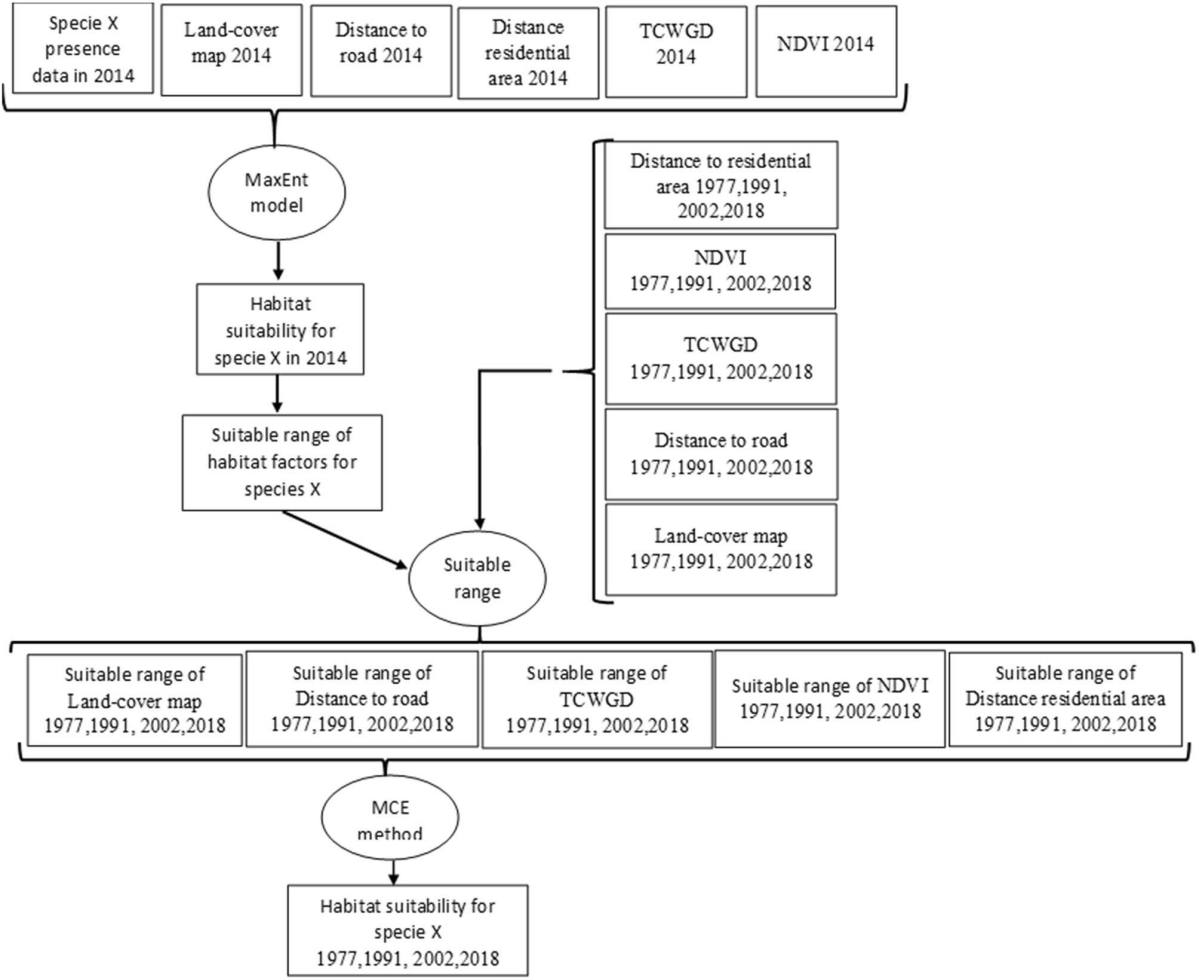
The flowchart of habitat modelling for one species (x). These steps were repeated for each species in Table 4. Then the suitable habitat maps for all species were overlaid to create the suitable habitat map for water birds. The priority to overlay the habitat suitability classes was based on the number of birds.

To determine suitable ranges of all habitat factors for water birds in the Hamoun wetlands, the habitat suitability map in 2014 was created, and the threshold of suitable ranges was achieved. Since the wetlands were dry in 2018, the water bird’s habitat in May 2014 was used to determine the suitable range of habitat factors in this area. Then, these ranges were used to map the habitat suitability map of other years of the study period (1977, 1991, 2002, 2018). Figure 9 shows the flowchart of creating the habitat maps. The suitable habitat maps for all species in Table 4 were overlaid to create the suitable habitat map for water birds (Fig. 9). The priority to overlay the habitat suitability classes was based on the number of birds. The details of creating habitat map are described below.

The water birds’ habitat suitability map of 2014 was created using the maximum entropy model (MaxEnt)^49^. The water birds’ presence data in 2014 were achieved from the previous study by Maleki et al.^43^. The ecological maps as input layers in the MaxEnt model included land-cover map, distance to road and residential area, normalized difference vegetation index (NDVI) map, and tasseled cap wetness-greenness difference (TCWGD) map. Distance to residential areas and roads, and the land-cover map were selected as factors influencing shelters. NDVI and TCWGD showed the availability of food and water. The correlation among variables was tested to eliminate highly correlated variables (r ≥ 0.85 Pearson correlation coefficient). Since there was no significant correlation among the variables, all of them were used in creating the habitat suitability map. The MaxEnt model was run by122 points of water birds’ presence points that 70% of the data were applied as training points, and the remaining points were used as tests. The result of this model is a continuous map. The MaxEnt Software provides threshold values for suitable classes based on various statistical measures: 10 percentile training presence logistic threshold. This is a threshold ignoring all regions with suitability lower than the suitability of 10% of occurrence records^50^. The suitable area on the produced map was divided into two levels: Suitable 1 and Suitable 2. The suitable 1 illustrates the best habitat for water birds in the Hamoun wetlands, and the class 2 is a moderate habitat for water birds in this area.

Based on the satellite images of 2002 and 2018, the wetlands were dry in these two years. Hence, there is no suitable habitat for water birds in 2002 and 2018. The habitat suitability maps in 1977 and 1991 were created using the multi-criteria evaluation method (MCE). Land-use/land-cover, distance to road and residential area, normalized difference vegetation index (NDVI) and tasseled cap wetness-greenness difference (TCWGD) maps were created using the satellite images. Suitable ranges of these factors in 1977 and 1991 were extracted based on their suitable ranges in 2014. Equation (2) was used to create the habitat suitability for 1977 and 1991.

2 $${\text{Mi}} = \mathop \sum \limits_{j = 1}^{n} \;{\text{Wj}} \times {\text{Xij}}$$

where Mi is the overall score of the habitat suitability for the *i*th gird cell. Wj is the weight of the *j*th variable (land-cover map, distance to road and residential area, NDVI map and TCWGD map). Xij is the value of the *i*th gird cell for the *j*th variable.

### Change analysis

**Figure 10:**
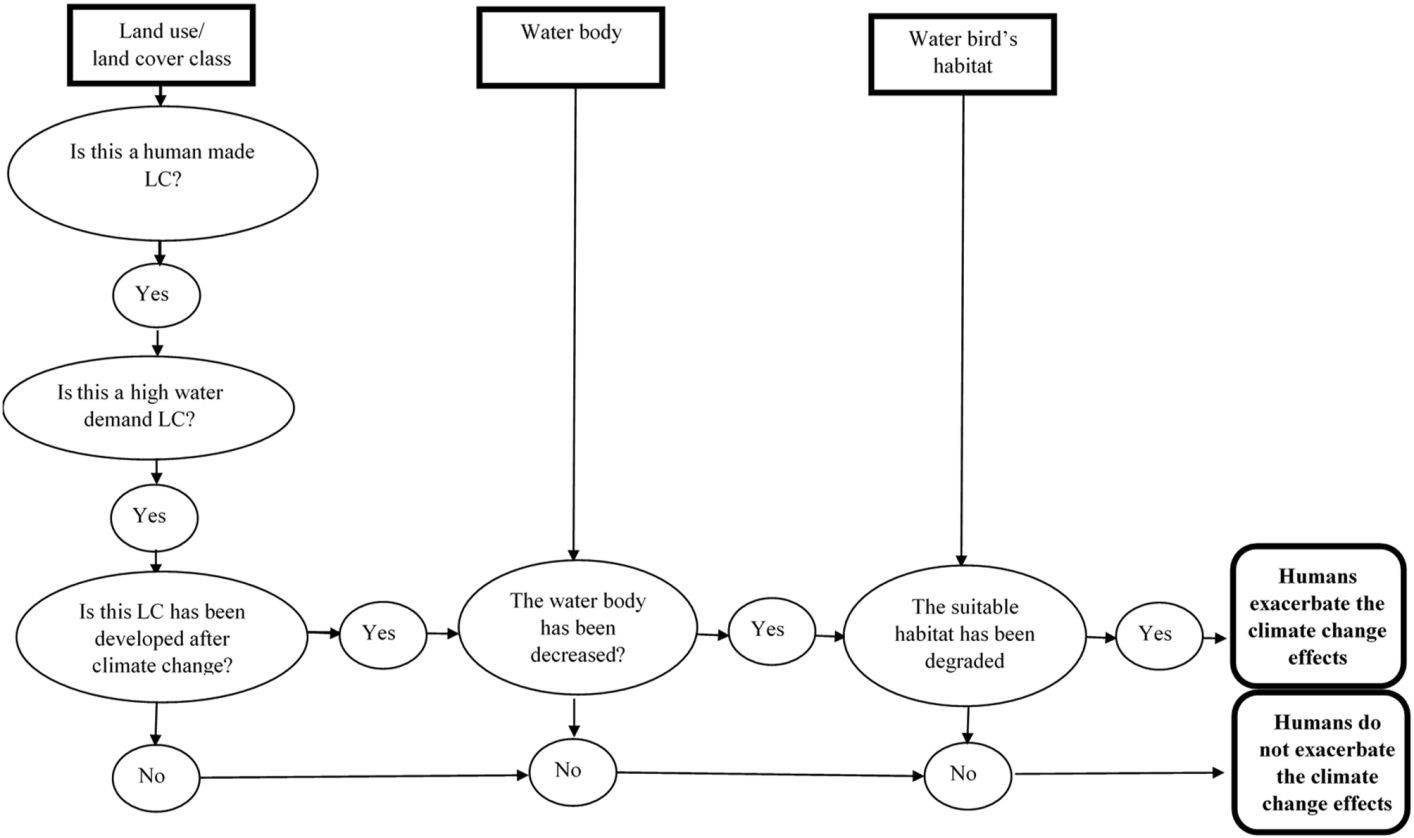
Change analysis flowchart.

To determine whether the human activities increase or decrease the negative effects of climate change, we followed the assumptions below based on a decision tree method using if–then logic (Fig. 10):

If land-use/land-cover is changed in accordance with the water limitation and habitat suitability alteration, humans do not exacerbate the climate change effects.

If land-use/land-cover classes with high water demand are developed in contrast to water limitation and water birds’ habitat suitability, humans exacerbate the climate change effects.
